# Pollutant effects on genotoxic parameters and tumor-associated protein levels in adults: a cross sectional study

**DOI:** 10.1186/1476-069X-7-26

**Published:** 2008-06-03

**Authors:** Sam De Coster, Gudrun Koppen, Marc Bracke, Carmen Schroijen, Elly Den Hond, Vera Nelen, Els Van de Mieroop, Liesbeth Bruckers, Maaike Bilau, Willy Baeyens, Greet Schoeters, Nik van Larebeke

**Affiliations:** 1 Study Centre for Carcinogenesis and Primary Prevention of Cancer, Department of Radiotherapy, Nuclear Medicine, and Experimental Cancerology, Ghent University Hospital, De Pintelaan 185 3K3, 9000 Ghent, Belgium; 2 Environmental toxicology, Flemish Institute of Technological Research (VITO), Boeretang 200, 2400 Mol, Belgium; 3 Laboratory of Experimental Cancerology, Department of Radiotherapy, Nuclear Medicine, and Experimental Cancerology, Ghent University Hospital, De Pintelaan 185 P7, 9000 Ghent, Ghent, Belgium; 4 Vrije Universiteit Brussel (VUB), Analytical and Environmental Chemistry (ANCH), Pleinlaan 2, 1050 Brussels, Belgium; 5 Provincial Institute of Hygiene, Kronenburgstraat 45, 2000 Antwerp, Belgium; 6 University of Hasselt, University Campus, Building D, 3590 Diepenbeek, Belgium; 7 Ghent University, Department of Public Health, UZ 2 Blok A, De Pintelaan 185, 9000 Ghent, Belgium

## Abstract

**Background:**

This study intended to investigate whether residence in areas polluted by heavy industry, waste incineration, a high density of traffic and housing or intensive use of pesticides, could contribute to the high incidence of cancer observed in Flanders.

**Methods:**

Subjects were 1583 residents aged 50–65 from 9 areas with different types of pollution. Cadmium, lead, p,p'-DDE, hexachlorobenzene, PCBs and dioxin-like activity (Calux test) were measured in blood, and cadmium, t,t'-muconic acid and 1-hydroxypyrene in urine. Effect biomarkers were prostate specific antigen, carcinoembryonic antigen and p53 protein serum levels, number of micronuclei per 1000 binucleated peripheral blood cells, DNA damage (comet assay) in peripheral blood cells and 8-hydroxy-deoxyguanosine in urine. Confounding factors were taken into account.

**Results:**

Overall significant differences between areas were found for carcinoembryonic antigen, micronuclei, 8-hydroxy-deoxyguanosine and DNA damage. Compared to a rural area with mainly fruit production, effect biomarkers were often significantly elevated around waste incinerators, in the cities of Antwerp and Ghent, in industrial areas and also in other rural areas. Within an industrial area DNA strand break levels were almost three times higher close to industrial installations than 5 kilometres upwind of the main industrial installations (p < 0.0001). Positive exposure-effect relationships were found for carcinoembryonic antigen (urinary cadmium, t,t'-muconic acid, 1-hydroxypyrene and blood lead), micronuclei (PCB118), DNA damage (PCB118) and 8-hydroxy-deoxyguanosine (t,t'-muconic acid, 1-hydroxypyrene). Also, we found significant associations between values of PSA above the p90 and higher values of urinary cadmium, between values of p53 above the p90 and higher serum levels of p,p'-DDE, hexachlorobenzene and marker PCBs (PCB 138, 153 and 180) and between serum levels of p,p'-DDE above the p90 and higher serum values of carcinoembryonic antigen. Significant associations were also found between effect biomarkers and occupational or lifestyle parameters.

**Conclusion:**

Levels of internal exposure, and residence near waste incinerators, in cities, or close to important industries, but not in areas with intensive use of pesticides, showed positive correlations with biomarkers associated with carcinogenesis and thus probably contribute to risk of cancer. In some rural areas, the levels of these biomarkers were not lower than in the rest of Flanders.

## Background

Flanders is one of the most densely populated areas in Europe, with intensive traffic, industrial activities and intensive farming close to habitation. The pilot Flemish Environment and Health Survey (FLEHS) showed differences in internal exposure to pollutants in function of area of residence and indicated that small differences in pollutant levels were associated with observable differences in effects [1-4]. These results entailed a larger-scale, five year (2002–2006) biomonitoring program on neonates, adolescents and older adults by the Flemish Centre for Environment and Health.

This program comprised measurements of internal exposure on each of these age groups. Concerning the neonates, follow-up studies concerning neuropsychic development, asthma and allergy were performed. Concerning the adolescents, effect biomonitoring comprised measurements of hormone levels in boys and sexual maturation of boys and girls. For adults, effect biomonitoring entailed genotoxic tests and measurements of tumor associated protein levels (reported in the present paper) and also measurements of the expression of selected genes. Also, for both adolescents and older adults a study was performed on the relationship between carcinogenesis-related biological effects and 36 polymorphisms in 23 genes involved in xenobiotic metabolism, DNA repair and oxidative stress. This biomonitoring program (2002–2006) already resulted in several publications: internal exposure to pollutants in adolescents was described by Schroijen *et al*. [5]; dietary exposure to dioxin-like compounds in adolescents, mothers aged 18 to 44 years, and adults aged 50 to 65 years was reported by Bilau *et al*. [6]; the association of thyroid hormone concentrations with levels of organochlorine compounds in cord blood of neonates was reported by Maervoet *et al*. [7]. A detailed report on the internal exposure to pollutants of the adults participating in the study reported here will be published elsewhere. All public information on the project, as well as an overview of these data on internal exposure, is already available on a website [8]. Several publications on yet unpublished results are in preparation.

Here we report our observations on cancer-related markers of biological effects in 50 to 65 year old adults. Because of the important role of somatic mutations in carcinogenesis [9], we included effect biomarkers for genotoxicity: oxidative DNA damage measured through a metabolite in the urine (8-hydroxy-deoxyguanosine), DNA strand breaks (comet assay), and micronuclei measured in peripheral blood cells. As we did previously [4], we also included tumor-associated protein levels measured in blood. In our present study we measured prostate-specific antigen (PSA), carcinoembryonic antigen (CEA) and p53 levels (p53) in serum. During the long latency period after initiation of carcinogenesis and/or under the impact of tumor promoters, some cells in the body might express certain aspects of the tumoral phenotype, which may result in the release, in body fluids, of macromolecules associated with this phenotype. An increased concentration of such molecules in body fluids might, to some extent, reflect a higher exposure to cancer-inducing or cancer-promoting agents [10-14] or an increased risk of cancer [15-22]. Using these biomarkers associated with carcinogenesis or with risk of cancer we aimed at investigating whether residence in Flemish areas with specific types and levels of pollution, in particular stemming from heavy industry, waste incineration, a high density of traffic and housing or intensive use of pesticides, could contribute to cancer risk.

Our project also intended to test the hypothesis that low levels (such as these occurring in the general population in Flanders) of internal exposure to pollutants known or suspected to cause cancer were associated with increases in levels of oxidative DNA damage, of DNA strand breaks, of micronuclei or of tumor associated proteins.

## Methods

### Selection of study areas

As areas with a high level of pollution stemming from heavy industry, the port areas of Antwerp ('Antwerp port') and Ghent ('Ghent port'), and the 'Albert canal' and 'Olen' industrial basins were chosen. At the start of the project the ports of Antwerp and Ghent were considered together as one industrial zone, but in view of differences in type of industry and in view of the results obtained (e.g. for the adolescents [5]), we thought it adequate to consider the results for the ports separately. 'Antwerp port' is an important industrial site with huge petrochemical industries, chemical and plastic industry and production of pesticides (n = 163 adults between 50–65 years old recruited in this area). However, only part of the participants from 'Antwerp port', such as those from Burcht, resided in zones that have an important exposure to industrial emissions, whereas others, from the municipality of Beveren, resided at a distance of about 6 kilometers to the west of the main industrial installations. 'Ghent port' has mainly metallurgic industries, however, all adult participants (n = 36) resided in the municipality of Evergem at the south west of the main industrial installations. 'Albert canal' is an industrial zone with chemical and plastic industries and production of electricity amidst rural areas (n = 196). 'Olen' is an industrial zone with a large non-ferrous smelter, and chemical, plastic and automobile industry amidst rural areas (n = 203). As areas with a high level of pollution pressure stemming from a high density of traffic and housing, the cities of Antwerp and Ghent were chosen. Antwerp, the largest city in Flanders, is an industrial city with 404,000 inhabitants and very dense traffic (n = 197). Ghent, the second largest city in Flanders, is a smaller industrial city with 213,000 inhabitants (n = 198). As an area with a high level of pollution pressure stemming from intensive use of pesticides, the 'fruit area' around Sint-Truiden was chosen, comprising a rural region with intensive apple or pear cultivation (n = 193). As area with a high level of pollution pressure stemming from waste incineration, neighborhoods close to waste incinerators in 6 municipalities, spread out over the whole of Flanders (n = 198) were chosen. These neighborhoods comprised a limited area, with a mean surface of 6.2 km^2 ^mainly under the wind of a waste incinerator. For comparison we included 'rural areas'. These rural areas are, in Flanders, certainly not devoid of environmental pollution and might (see discussion) even show some higher exposures [5,23] due to certain local habits such as burning waste, inappropriate use of pesticides and consumption of self-grown food [24]. However, with a lower population density, less traffic and no local heavy industry they constitute an interesting point of comparison. In our study 'rural areas' comprised 24 municipalities, spread out over 9 contiguous areas in the western half of Flanders with no highways and no industries reported in the emission inventory of the Flemish environmental protection agency (n = 199).

**Figure 1:**
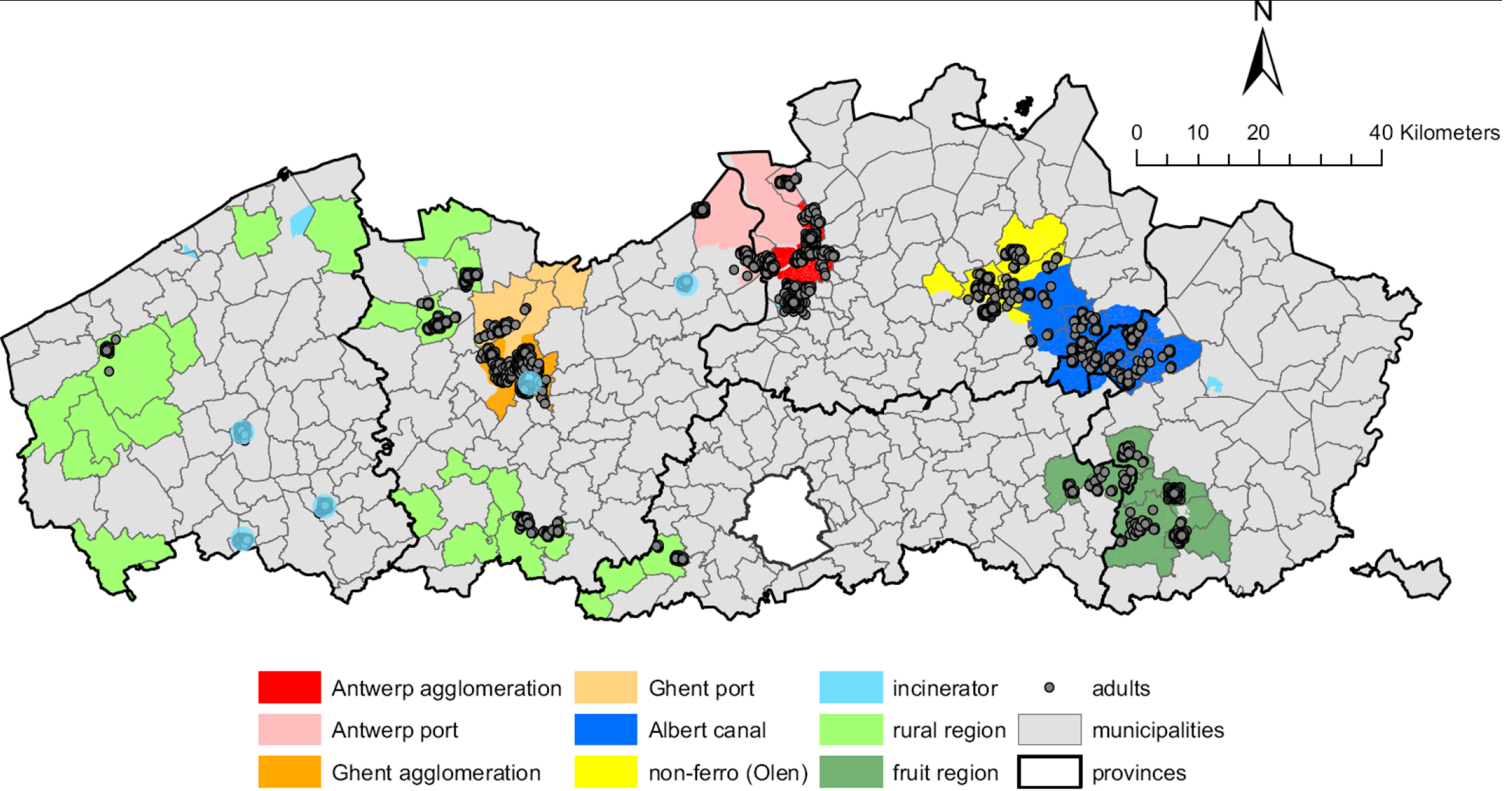
Map of the 9 areas in which participants for the biomonitoring program were recruited.

Table 1 summarizes characteristics, including some emission data, of the different study areas, and figure 1 shows their position in Flanders. The surface of the studied area is 3,036 km^2^, corresponding to 22% of the total surface of Flanders (13,521 km^2^). The 65 selected municipalities correspond to 20% of the total Flemish municipalities. Except for 'rural Flanders' and for 'waste incinerators', all study areas were contiguous geographical entities.

**Table 1:** Characteristics and emission data for the 9 study areas

**Area of interest**	**Surface (km^2^)**	Number of inhabitants*	**Characteristics and sources of pollution**	Pesticide use (kg/km^2^)**	Industrial Emission to air (per year)***	Industrial Emission to surface water (per year)***
Antwerp	81	404,241	Metallurgic industry, large non-ferrous smelter, important highways, huge traffic	13.3	80 kg PAH	
Antwerp Port	179	64,510	Huge petrochemical industries, chemical and plastic industry, production of pesticides	117	15 kg Cd	15 kg Pb
					837.5 kg PAH	4.03 kg Cd
					93,729 kg benzene	4.1 kg pesticides
						3.2 kg PAH
						640.9 kg benzene
Fruit area	362	95,829	Apple and pear orchards: more than 10 hectares per km^2^	617		
Olen	183	68,068	Large non-ferrous smelter, delineated in function of modelled, calculated immission of at least 0.9 ng lead per m^3 ^from the non-ferrous smelter. Chemical, plastic and automobile industry. Rural areas	34.6	810 kg Pb	88 kg Pb
					87 kg Cd	39.71 kg Cd
					4,050 kg benzene	
Ghent	100	213,025	Metallurgic and automobile industry, intensive traffic	27.7	150 kg Pb	24.7 kg Pb
Waste incinerators	37	56,405	Waste water and waste treatment. Delineated in function of modelled, calculated immission of at least 1.2 mg smoke per m^3 ^from the waste incinerator	80.1	14 kg Cd	48.7 kg Pb
						1.89 kg PAH
Rural area	1181	153,770	Less than 250 inhabitants per km^2^. No highways crossing the municipalities. No industries reported in the emission inventory of the Flemish Environmental protection agency.	233		
Albert canal	711	64,763	Chemical and plastic industries, rural areas, production of electricity	20.8	430 kg benzene	59.5 kg Pb
						67.36 kg Cd
Ghent Port	202	65,554	Mainly metallurgic industries	99.2	34,500 kg Pb	1,690 kg Pb
					542.1 kg Cd	
					1,823 kg PAH	
					225 kg benzene	

**Total**	**3036**	**1,186,165**			**35,460 kg Pb**	**1,925.9 kg Pb**
					**658,1 kg Cd**	**111.1 kg Cd**
					**2,740.5 kg PAH**	**4.1 kg pesticides**
					**98,434 kg benzene**	**5.09 kg PAH**
						**640.9 kg benzene**

* Based on the number of inhabitants of 1998; ** Belgian ministry of Economic affairs and Institute for Social and Economic Geography, Catholic University of Leuven; *** Emissions of pollutants reported by the emitting companies themselves to the Flemish Environmental Protection Agency .

### Selection and recruitment of participants

A Stratified Clustered Multi-Stage Design was used to select 775 men and 808 women (n = 1583) aged 50 to 65 as a random sample of the population of the areas under study. In the selected areas approximately 1.2 million inhabitants are living which is 20% of the Flemish population. A sample size of 200 participants per study area was aimed at because a power calculation demonstrated that this appears to be statistically sufficient to detect differences of 20% in internal pollutant concentrations between study areas. As the port area was further split in two different study areas, the sample size was lower in the individual port areas of Antwerp (n = 163) and Ghent (n = 36). Sampling took place in three steps: first by study area, secondly by sub-municipality entities for access to participants, and thirdly by selection of the participants in accordance with the inclusion criteria.

### Personal characteristics and sampling

Height and weight were measured, body mass index (BMI) was calculated. 200 mL of urine and 40 mL of blood were collected from each participant. Immediately after sampling, serum was separated. Samples of serum, whole blood, and urine were stored at 4°C for maximum one week, or immediately deep frozen. All laboratory analyses were performed, using blinded methods, in specialized laboratories that met national and international quality-control standards.

### Information from questionnaires

A self administered questionnaire was used to collect information on physical condition, use of medication, education, professional exposure to various pollutants (solvents, metals, polycyclic aromatic hydrocarbons [PAHs], asbestos, radioactive and non-ionizing electromagnetic radiation, halogenated hydrocarbons and reactive substances), housing conditions, pesticide use, exposure to noise and traffic, quality and quantity of homegrown, local and other food consumption, alcohol consumption and smoking. On the basis of these questionnaires a number of parameters were calculated, including average amount consumed per day of fruit, vegetables, meat fat, fish fat and dairy fat. Other parameters concerned self-caught freshwater fish, recent and lifetime tobacco consumption, recent and average alcohol consumption, and indices for general indoor housing quality, exposure to traffic, to indoor and outdoor stoking of diverse organic materials and for experiencing nuisance from noise during the day and during the night. Smoking was quantified in terms of units (cigarettes, cigars, pipes) smoked during the entire lifetime. For people 50–65 years of age, 15 cigarettes a day corresponds roughly to 200.000 units over a lifetime.

### Measured internal exposure

Measured exposures included blood cadmium (μg Cd/l), urinary cadmium (μg Cd/g creatinin), blood lead (μg/l), serum hexachlorobenzene (ng HCB/g lipid), serum p,p'-DDE (ng/g lipid); serum polychlorinated biphenyls (PCBs): PCB 99, PCB 118, PCB 170, PCB 138, PCB 153, PCB 180 (ng/g lipid); serum Calux TEQ (toxic equivalents, pg PCDD/F Calux TEQ/g lipid); a biomarker for PAH exposure, 1-hydroxypyrene in urine (1-OHP, μg/g creatinine); and a biomarker for benzene, t,t'-muconic acid in urine (ttMA, mg/g creatinine).

Lead and cadmium concentrations in whole blood were determined after an acid digestion pre-treatment destroying the organic matrix and a ten times dilution, followed by High Resolution – Inductively Coupled Plasma – Mass Spectrometry detection (ICP-MS) as described by Schroijen *et al*. [5]. Detection limits for cadmium and lead in whole blood were 0.09 and 2.0 μg/L respectively for digested blood samples diluted 10 times. Isotope Cd114 was used to quantify the amount of cadmium in urine using ICP-MS. Urine samples were diluted in nitric acid (0.7%). Rhodium was used as an internal standard. The detection limit for urinary cadmium was 0.002 ppb.

PCBs, HCB and p,p'-DDE were analyzed using gas-chromatography/electron capture detection (GC-ECD) [25]. 2 mL of blood serum was mixed with 2.85 mL formic acid and 150 μL acetonitrile. Internal standards (1 ng each of PCB 143 and PCB 207) were added and the mixture was equilibrated in an ultrasonic bath for 20 min. The sample was eluted through an Oasis cartridge at atmospheric pressure. Subsequently the cartridge was washed with 5 mL methanol/water (1:19) and dried. Elution of the analytes was performed with 3 mL toluene. The toluene extract was purified using a multilayer column containing (top to bottom) 200 mg anhydrous sodium sulphate, 400 mg silica modified with sulphuric acid (44% w/w) and 400 mg silica. The final extract was concentrated under a stream of nitrogen to a volume of 100 μL and 0.6 ng of 1,2,3,4-tetrachloronaphthalene was added to quantify the recovery of the internal standards. The extracts were analyzed with GC-ECD. The detection limit of all chlorinated compounds in serum was 0.02 μg/L.

1-Hydroxypyrene (a metabolite of pyrene, 1-OHP), and t,t'-muconic acid (a metabolite of benzene, ttMA) were detected in urine based on methods used by Angerer & Schaller [26]. The determination of 1-OHP was performed with High Performance Liquid Chromatography (HPLC). To release 1-OHP from proteins, urine was hydrolysed by the enzymes β-Glucuronidase and Arylsulfatase (Roche, Belgium) during the night. Then 1-OHP was on-line extracted from the urine matrix and enriched and separated on an apolar C-18 reversed phase column with a gradient solvent mixture of methanol and water. The detection limit and quantification limit were 0.030 μg/L and 0.092 μg/L, respectively. t,t'-Muconic acid was determined in urine by means of ion chromatography using SPE-SAX columns. HPLC, with a solvent gradient of methanol and acetic acid (1% v/v), was used to separate the extract from other compounds. The detection limit and quantification limit were 0.0086 mg/L and 0.0213 mg/L, respectively.

CALUX analyses of dioxin-like activity in blood plasma was performed as described by Van Wouwe *et al*. [27] and Schroijen *et al*. [28]. Briefly, 5 mL of blood plasma was extracted with acetone and *n*-hexane and dried on a Celite/Na_2_SO_4_column. The extract was then transferred on an Acid Silica column in series with an activated Carbon column (XCARB column). After elution of the sample with *n*-hexane, the acid silica column was discarded and the XCARB column was then differentially eluted to yield 3 fractions:

1. a mixture of *n*-hexane/acetone allows the elution of some toxic or interfering compounds

2. the PCB fraction is eluted with a mixture of *n-*hexane/toluene/ethyl acetate

3. the PCDD/F fraction is collected with 20 mL of toluene.

After this clean-up, fractions 1 and 2 were discarded and only the dioxin fraction was used for the bio-analysis. The solution containing the dioxins was then evaporated and exposed to the mouse hepatoma H1L6.1 cell line developed by Xenobiotic Detection System, Inc. After an exposure time of 20 h, cells were lysed and measurements were made with a luminometer. TEQ-values were calculated after comparison of the obtained signals to a 2,3,7,8-TCDD calibration curve.

### Selection of biomarkers of effects

The following is a summary of the data on which our selection of biomarkers of effect was based.

Increased serum levels of Prostate Specific Antigen (PSA) are found in the vast majority of men with prostate cancer. A Finnish study found, for men under 65 years of age, a sensitivity of 93% and a specificity of 96% for PSA as a diagnostic test for prostate cancer using a limit value of 4 ng/mL [29]. Serum PSA level also allows to assess risk of prostate cancer. Of men with a normal PSA level of 2.1 to 4.0 ng/mL, 1.6% to 5.5% were diagnosed with prostate cancer within one year, whereas only 0.06% to 1.02% of men with a PSA level of 1.1–2.0 ng/mL did so and only 0.01% of men with a PSA level lower than 1 ng/mL was diagnosed with prostate cancer in a period of three years [30]. Increased serum PSA levels were found in men exposed to various pollutants or toxic substances (see discussion).

Serum levels of Carcinoembryonic Antigen (CEA) are increased in many types of cancer, in smokers and in persons exposed occupationally or environmentally to pollutants [31-33]. Serum CEA levels measured years before clinical diagnosis showed a positive correlation with risk of cancer [34,35]. In our pilot biomonitoring study [4] we found a positive correlation between CEA serum levels on the one hand and blood lead levels, an index reflecting internal exposure to several pollutants, and HPRT mutant frequency in peripheral blood cells on the other.

Increased serum levels of mutated p53, or of anti-p53 antibody, have been found in cancer patients [36] and in workers exposed to carcinogenic substances [10,13,14,37,38]. Anti-p53 antibodies or increased levels of mutant p53 protein can predate the diagnosis of cancer [39-41]. In our pilot biomonitoring study we found a positive correlation between anti-p53 antibody levels on the one hand and blood lead levels and an index reflecting internal exposure to several pollutants on the other. At the start of the project we gave preference to p53 serum level above anti-p53 antibody serum level, because it was reported that increased levels of p53 would occur in more persons than increased levels of anti-p53 antibodies [10].

Measurement of the number of micronuclei per 1000 binucleated peripheral blood cells (micronuclei) is one of the best established biomarkers of chromosome damage [42]. An increased frequency of micronucleated cells is a biomarker of genotoxic effects that can reflect exposure to agents with clastogenic (chromosome breaking; DNA as target) or aneugenic (aneuploidogenic; effect on chromosome number; mostly non-DNA target) modes of action [43]. An increased micronucleus frequency in peripheral blood lymphocytes predicts the risk of cancer in humans [44]. This test reflects genetic damage that occurred over a longer period of time and is relatively stable over a 12 month period [45]. Sensitivity is rather low. We estimated that a difference of the order of 30% is needed in order for the test to reach a power of the order of 0.9 in discriminating between two groups of 100 persons [45]. The micronucleus test is simpler, cheaper and is less time consuming than the chromosome aberration assay that was too expensive in the context of our project.

Measurement of DNA-strand breaks in peripheral blood cells in terms of the median value of the percentage of DNA in the tail using the comet assay was selected to reflect recent damage to DNA. The alkaline single-cell gel electrophoresis (SCGE) or Comet assay is an easy, reliable and rapid method to detect DNA single strand breaks, alkaline labile sites and delayed repair sites of DNA. It is able to detect low levels of DNA damage, requires small sample sizes and has a rather low cost [46]. The inter and intra-assay coefficients of variation are of the order of 20% [47]. We estimated that the assay has a power of the order of 0.90 to discriminate between two groups of about 30 persons showing a mean difference of 10% [46].

The measurement of 8-hydroxy-deoxy-guanosine (HDG) in urine per gram creatinine was selected to assess recent oxidative stress. HDG is excreted in the urine after excision of this mutagenic oxidative adduct from DNA and reflects the amount of oxidative damage to DNA and its repair. Its measurement is affordable and has inter- and intra-assay coefficients of variation of 8 to 24% [48]. We estimated that the test has a power of the order of 0.9 to discriminate between two groups of about 100 persons showing a mean difference of 12% [46].

### Biomarkers of effects: methods of measurement

Tumor associated protein levels in serum were measured by Interlab, (Evergem, Belgium) a laboratory recognized by the Belgian Ministry of Health as a reference laboratory for tumor markers. CEA and PSA serum concentrations were both measured with an immunometric assay. The Immulite^® ^2000 (DPC, Los Angeles, CA, USA) technology was applied on 15 and 10 μl serum samples respectively, that were assessed within 24 h after venipuncture. The within-run precisions of the methods applied on samples in the normal, non-pathological range were 3.0 and 3.3% (coefficients of variation) respectively. p53 levels were analysed in 100 μL serum samples with the TiterZyme EIA p53 Kit (Assay Designs, Inc., Ann Arbor, MI, USA) in accordance with the manufacturer's instructions. The lower limit of detection (analytical sensitivity) was determined on the basis of the mean of the zero standard (procedural blank) plus two standard deviations, and calculated to be 9 pg/mL. The monoclonal capture antibody recognizes both wild-type and mutant human p53. p53 results below the detection limit were given the value of 4.5 pg/ml.

The amount of DNA-strand breaks was evaluated by the alkaline comet assay as described in Verschaeve *et al*. [49]. The whole blood cells were kept at room temperature during maximum 2 days after the blood was taken. The comet assay methodology consisted of embedding the whole blood cells in agarose and layering on microscope slides. After lysis of the nuclei, the slides were put for 40 min in a horizontal electrophoresis chamber filled with alkaline buffer to allow unwinding of the DNA. Electrophoresis in this buffer (20 min, at 1 V/cm) was followed by washing and staining with ethidium bromide. The slides were viewed using the image analysis system Methasystems Finder, version 2.8.0^®^, Methasystems inc. For each person 30% of the slide was viewed. Around 200 cells were processed and the median percent of DNA migration in the tail area was determined and used as a measure of DNA damage. As positive control one slide with nuclei from deep frozen whole blood cells was added to each electrophoresis chamber. In those positive controls at least 30% of DNA had to migrate into the tail area to control the functioning of the electrophoresis.

The cytokinesis-block micronucleus assay was performed on whole blood cultures from 100 individuals using standard procedures [50]. For each individual, 1000 binucleated cells were evaluated for the presence of micronuclei on a Zeiss Axioplan microscope with a 100× objective.

Urinary HDG, a measure for oxidative DNA-damage, was measured via ELISA using the competitive inmunosorbent assay-kit (Gentaur, Belgium).

### Statistical analyses

Statistical analyses were performed with the Statistica 7.1 program (Statsoft, Tulsa, OK, USA). The effect parameters did not show a Gaussian distribution, so the natural logarithm of these parameters was used in statistical analysis. Crude data (median and p10 and p90) as well as adjusted data are shown. As we did in our previous studies [5,23], we used the parameter 'marker PCBs' (the sum of serum concentrations of PCBs occurring in the highest concentrations: PCB 138 + PCB 153 + PCB 180) in our analysis to reflect exposure to PCBs in general whereas the other PCBs were considered individually. Confounding factors were taken into account. A confounding factor is a variable that correlates with both the dependent and the independent variable and controlling for confounding is necessary to avoid false positive conclusions that the dependent variable is correlated with the independent variable. Differences between areas after correction for confounding factors were evaluated by means of Analysis of Covariance (ANCOVA). Confounding factors for analyses were defined as age, sex, body mass index (BMI, for analysis including PSA, PCBs, HCB and p,p'-DDE) and lifetime smoking (PSA, CEA, p53, micronuclei) or recent smoking (DNA-strand breaks, oxidative DNA damage). When the 9 areas showed an overall significant difference in ANOVA, then the mean value of each study area was compared with the area showing the lowest mean value using the post hoc Fisher protected least significant difference (PLSD) test.

Also, effect biomarker levels of each area were compared to the rest of the dataset. The correlation of biomarkers of effect (as dependent variables) with the measured parameters of internal exposure and with some exposure variables derived from questionnaires was studied in simple linear regression. When this analysis resulted in a p-value below 0.05, a multiple regression was performed with the biomarker of effect as dependent variable and, as independent variables, in addition to the parameter of exposure in question, also the relevant confounding factors.

Some sets of effect and exposure biomarkers showed, in a bivariate regression plot, contrasting correlations for different ranges of exposure or effect. In those cases we used p10, p25–p75 and p90 levels to discriminate classes of exposure or effect in order to study correlations in the different ranges using AN(C)OVA.

We calculated for each biomarker of exposure a standard or z score for each individual by dividing the difference between the value for that individual and the mean value for the entire subject population by the standard deviation for the entire subject population. We calculated for each subject an index of internal exposure I_ex _defined as the arithmetic mean of the z scores for blood lead concentration, sum of serum concentrations of marker PCBs (138, 153 and 180), serum concentration of PCB 118 (considered to reflect exposure to dioxin-like PCBs [51]), HCB and p,p'-DDE, dioxin-like activity in serum measured through the calux bioassay, urinary excretion of cadmium, 1-hydroxypyrene and of t-t'-muconic acid per g creatinin (I_ex _= (z_blood lead _+ z_sum of serum marker PCbs _+ z_PCB118_+ z_HCB _+ z_DDE _+ z_dioxin-like activity _+ z_urinary Cd _+ z_1-OHP _+ z_ttMA_)/9).

## Results

### Range of internal exposure

Table 2 shows crude data concerning measured internal exposure in the whole study population. A detailed report on the impact of area of residence and of other factors on the internal exposure will be published elsewhere.

**Table 2:** Crude biomarkers of exposure for the whole study area

**Biomarker of exposure**	**n**	**median**	**p10**	**p90**
Cadmium blood (μg/L)	1,579	0.48	0.16	1.24
Cadmium urine (mg/g crt)	1,581	0.62	0.32	1.30
Lead (μg/L)	1,579	39.18	20.18	76.35
HCB (ng/g fat)	1,577	55.92	28.17	121.36
PCB99 (ng/g fat)	1,577	10.81	1.66	24.35
PCB118 (ng/g fat)	1,577	25.85	11.71	50.68
PCB156 (ng/g fat)	1,577	18.90	11.01	31.89
PCB170 (ng/g fat)	1,577	40.28	24.91	64.41
Sum marker PCBs (138+153+180) (ng/g fat)	1,577	345.48	213.23	532.15
Calux assay (pg TEQ/g fat)	1,437	22.90	5.43	45.71
p,p'-DDE (ng/g lipid)	1,577	487.33	141.60	1,587.30
1-OHP (μg/g crt)	1,575	0.143	0.032	0.684
ttMA (mg/g crt)	1,391	0.082	0.017	0.334

Median (p10–p90) crude values and number of cases.

### Differences in biomarkers of effect between main areas

Table 3 shows crude data, number of cases (n) and p-values in ANCOVA (without correction for confounding) and table 4 shows adjusted data and p-values in ANCOVA after correction for confounding factors, for seven biomarkers that were measured in the study population in the nine different areas considered in Flanders.

**Table 3:** Effect-biomarkers for different main study areas: crude values.

**Main area**		**PSA (ng/mL) p = 0.23**	**CEA (ng/mL) p = 0.014**	**p53 (pg/mL) p = 0.33**	**Micronuclei (number per 1000 binucleated cells) p = 0.15**	**DNA-strand breaks (comet assay, %DNA) p = 0.00015**	**Oxidative DNA damage (HDG μg/g crt) p = 0.05**
Antwerp	median	1.17	1.91***(H)	4.5	7.30	1.69	14.5
	p10, p90	0.39, 3.31	**0.88, 4.70**	4.5, 109.0	2.70, 14.90	0.55, 3.39	9.2, 22.3
	n	97	**125**	117	109	68	50
Antwerp Port	median	0.95	1.55	4.5	6.65	**1.23#**^a^**(L)**	15.9
	p10, p90	0.40, 3.53)	0.80, 3.94	4.5, 84.0	2.90, 13.30	**0.56, 4.67**	8.0, 21.8
	n	75	64	64	62	**49**	39
Fruit Area	median	0.88#	**1.57# (L)**	4.5	**6.00# (L)**	**1.35 (L)**	15.5*
	p10, p90	0.33, 2.17	**0.59, 3.60**	4.5, 161.0	**2.30, 14.10**	**0.85, 3.19**	11.4, 26.1
	n	100	**88**	111	**75**	**44**	35
Olen	median	0.97	1.57	4.5	7.00	1.60	14.3
	p10, p90	0.35, 3.42	0.72, 5.41	4.5, 121.0	3.00, 12.50	0.61, 2.60	8.2, 23.4
	n	95	79	79	79	74	40
Ghent	median	0.96	1.88*	4.5	7.25	2.03*** (H)	15.3
	p10, p90	0.45, 2.96	0.81, 4.30	4.5, 111.0	3.20, 14.30	**0.97, 3.65**	9.7, 22.9
	n	93	99	99	98	**85**	72
Waste Incinerators	median	0.86	1.89*	4.5#	**8.60 (H)**	2.24*** (H)	17.9** (H)
	p10, p90	0.30, 2.49	0.82, 4.33	4.5, 59.0	**2.90, 17.40**	**0.95, 3.13**	**9.4, 28.4**
	n	94	121	102	**101**	**100**	**51**
Rural area	median	1.06	1.79	4.5	7.00	1.97**	**14.7# (L)**
	p10, p90	0.34, 2.87	0.80, 3.31	4.5, 159.0	2.50, 16.10	1.03, 2.95	**8.0, 20.8**
	n	100	114	114	110	101	**76**
Albert Canal	median	1.08	**1.49 (L)**	4.5	7.10	1.97	15.5
	p10, p90	0.47, 3.17	**0.80, 3.30**	4.5, 139.0	3.00, 15.60	0.95, 2.67	10.0, 21.9
	n	97	**103**	103	103	37	35
Ghent Port	median	0.81	1.84	4.5	6.60	1.73	9.3
	p10, p90	0.43, 2.76	0.78, 4.08	4.5, 512.0	3.20, 14.20	1.00, 2.57	-
	n	19	36	36	36	35	1

**H **indicates significantly higher values for the area compared to the rest of Flanders.

**L **indicates significantly lower values for the area compared to the rest of Flanders.

# area with lowest crude geometric mean. Significant differences with the area with the lowest geometric mean in an LSD post hoc test are indicated by *p < 0.05, **p < 0.01, ***p < 0.001.

^a ^This low value is due to the residents of Beveren situated about 6 kilometres upwind of the industrial installations, who had a mean value of 1.10% (see text).

Median and p10–p90 crude values and number of cases. Statistical significance is assessed in ANOVA.

**Table 4:** Effect-biomarkers for different main study areas: adjusted values.

**Main area**		**PSA (ng/mL) p = 0.30**	**CEA (ng/mL) P = 0.034**	**p53 (pg/mL) p = 0.40**	**Micronuclei (number per 1000 binucleated cells) p = 0.045**	**DNA-strand breaks (comet assay, %DNA) p = 0.00017**	**Oxidative DNA damage (HDG μg/g crt) p = 0.037**
	*Confounding variables*	*A/LS/BMI*	*A/S/LS*	*A/S/LS*	*A/S/LS*	*A/S/RS*	*A/S/RS*

Antwerp	geometric mean	1.13	1.94***	9.3	6.64*	1.54	14.4
	95% CI	0.95, 1.34	1.75, 2.16	7.1, 12.2	5.86, 7.53	1.35, 1.75	13.0, 16.0
	n	97	125	117	109	68	50
Antwerp Port	geometric mean	0.92	1.63	10.4	5.95	**1.42# **^a ^**(L)**	14.7
	95% CI	0.76, 1.11	1.41, 1.88	7.3, 15.0	5.05, 7.02	**1.21, 1.66**	13.0, 16.5
	n	75	64	64	62	**49**	39
Fruit Area	geometric mean	**0.82# (L)**	**1.59 (L)**	11.7	**5.16# (L)**	**1.44 (L)**	16.6*
	95% CI	**0.70, 0.98**	**1.40, 1.80**	8.9, 15.4	**4.44, 6.00**	**1.22, 1.69**	14.7, 18.8
	n	**100**	**88**	111	**75**	**44**	35
Olen	geometric mean	1.02	1.77	10.8	6.57*	1.57	14.0
	95% CI	0.86, 1.21	1.55, 2.02	7.8, 14.9	5.67, 7.60	1.38, 1.79	12.5, 15.8
	n	95	79	79	79	74	40
Ghent	geometric mean	1.02	1.89*	11.3	6.77*	1.97*** (H)	15.1
	95% CI	0.86, 1.21	1.68, 2.12	8.5, 15.2	5.94, 7.72	**1.75, 2.22**	13.8, 16.4
	n	93	99	99	98	**85**	72
Waste	geometric mean	0.91	1.89**	9.2#	7.51*** (H)	2.03*** (H)	17.3** (H)
Incinerators	95% CI	0.77, 1.08	1.70, 2.10	6.9, 12.2	**6.60, 8.55**	**1.82, 2.26**	**15.6, 19.1**
	n	94	121	102	**101**	**100**	**51**
Rural area	geometric mean	0.94	1.85	13.3	6.75*	1.86**	14.0#
	95% CI	0.80, 1.11	1.65, 2.06	10.1, 17.4	5.97, 7.65	1.67, 2.07	12.8, 15.2
	n	100	114	114	110	101	76
Albert Canal	geometric mean	1.09	**1.54# (L)**	13.9	6.57*	1.66	15.0
	95% CI	0.92, 1.29	**1.37, 1.73**	10.5, 18.5	5.78, 7.46	1.39, 1.99	13.2, 16.9
	n	97	**103**	103	103	37	35
Ghent Port	geometric mean	1.00	1.91	13.53	6.44	1.66	8.9
	95% CI	0.68, 1.47	1.58, 2.32	8.4, 21.9	5.18, 8.00	1.38, 1.99	-
	n	19	36	36	36	35	1

**H **indicates significantly higher values for the area compared to the rest of Flanders.

**L **indicates significantly lower values for the area compared to the rest of Flanders.

# area with lowest adjusted geometric mean.

Significant differences with the area with the lowest geometric me an in an LSD post hoc test are indicated by *p < 0.05, **p < 0.01, ***p < 0.001.

^a ^This low value is due to the residents of Beveren situated about 6 kilometres upwind of the main industrial installations, who had a geometric mean value of 1.00% (see text below). A = age, S = sex, LS = lifetime smoking, RS = recent smoking, BMI = body mass index.

Adjusted geometric means, 95% confidence intervals and number of cases. Statistical significance is assessed in ANCOVA after adjustment for confounding factors as described in the text.

After correction for confounding factors, overall differences for effect biomarker levels between areas were observed for CEA, micronuclei, DNA-strand breaks and oxidative DNA damage (p = 0.034, 0.045, 0.00017 and 0.037 respectively). Compared to the rest of the dataset, significantly higher values of biomarkers of effect were observed for residents of 'waste incinerators' (micronuclei, DNA-strand breaks, oxidative DNA damage) and Ghent (DNA-strand breaks). Compared to the area with the lowest observed value, significantly higher values were observed for persons residing near waste incinerators (CEA, micronuclei, DNA-strand breaks, oxidative DNA damage), in Antwerp (CEA, micronuclei), in Ghent (CEA, micronuclei, DNA-strand breaks), in the 'rural area' (micronuclei, DNA-strand breaks), in the 'Albert canal' area (micronuclei), in the 'Olen' area (micronuclei) and in the 'fruit area' (oxidative DNA damage).

After additional correction for nutrition, alcohol consumption and education, overall significant differences between areas were still observed for oxidative DNA damage (p = 0.040) and for DNA-strand breaks (p < 0.001), but p-values increased for CEA (p = 0.16) and for micronuclei (p = 0.11).

### Differences between local districts within main areas

Within the main areas differences were observed (after correction for confounding) between small local districts. Between the areas around the different waste incinerators significant differences were observed for DNA-strand breaks (p < 0.0001) with values (adjusted geometric mean; 95% confidence interval; number of cases) of residents around the incinerators of Menen (2.19% DNA; 95% CI: 1.93, 2.50; n = 34), Roeselare (2.46% DNA; 95% CI: 2.15, 2.82; n = 31) and Wilrijk (1.89% DNA; 95% CI: 1.52, 2.36; n = 12) significantly higher than those of Harelbeke (1.34% DNA; 95% CI: 1.13, 1.59; n = 23). Within 'Antwerp port' a significant overall difference for DNA-strand breaks (p < 0.0001) was found, with values in Burcht, close to a large non-ferro industry (2.97% DNA; 95% CI: 2.23, 3.95; n = 14) significantly higher than Beveren situated about 6 kilometres upwind of the mean industrial installations (1.00% DNA; 95% CI: 0.83, 1.20; n = 34). For 'rural area' significant overall differences were found for CEA (p = 0.03) with significantly higher values (adjusted geometric mean; 95% confidence intervals; number of cases) in the rural areas around Brakel (2.03 ng/ml; 95% CI: 1.72, 2.40; n = 37) as opposed to those around Eeklo (1.63 ng/ml; 95%CI: 1.46, 1.83; n = 77).

### Associations of biomarkers of effect with levels of internal exposure: parameters showing an association over the whole range of measured values

Table 5 presents significant (p < 0.05) relationships, after correction for confounding factors, between measured exposure and effect biomarkers. Significant positive correlations have been found for CEA (urinary cadmium, lead, ttMA, 1-OHP, index of internal exposure I_ex_), Micronuclei (PCB118), DNA-strand breaks measured through the comet assay (PCB118) and oxidative DNA-damage assessed through measurement of HDG in urine (ttMA, 1-OHP).

**Table 5:** Associations between levels of biomarkers of effect and measured internal exposure.

**Effect biomarker**	**n**	**Parameter of internal exposure**	**Confounding factors**	**Regression coefficient**	**Standardized regression coefficient (95% CI)**	**Squared semi-partial correlation**	**p-value**
CEA (ng/mL) n = 829	829	Lead μg/L	A/S/LS	0.0084	0.104 (0.044, 0.165)	0.0136	< 0.001
		1-OHP μg/g crt	A/S/LS	0.427	0.101 (0.039, 0.164)	0.0120	0.0016
		Cadmium urine mg/g crt	A/S/LS	0.380	0.099 (0.034, 0.165)	0.0105	0.0032
		ttMA mg/g crt	A/S/LS	1.111	0.092 (0.030, 0.154)	0.0103	0.0036
		Index of internal exposure (I_ex_)	A/S/LS	0.445	0.101 (0.037, 0.166)	0.0090	0.0022
Micronuclei (number per 1000 binucleated cells)	773	PCB118 ng/g fat	A/S/LS/BMI	0.0280	0.092 (-0.095, 0.278)	0.0091	0.0083
DNA-strand breaks (%DNA, comet assay)	593	PCB118 ng/g fat	A/S/RS/BMI	0.0051	0.093 (0.007, 0.178)	0.0077	0.034
Oxidative DNA-damage (μg HDG/g crt)	399	ttMA mg/g crt	A/S/RS	4.047	0.096 (-0.003, 0.194)	0.0092	0.057
		1-OHP μg/g crt	A/S/RS	3.178	0.179 (0.077, 0.282)	0.0289	< 0.001

A multiple regression was performed with each effect biomarker as dependent variable with a measured exposure variable and confounding factors as independent variables. CI: confidence interval, n: number of cases, A = age, S = sex, LS = lifetime smoking, RS = recent smoking, BMI = body mass index.

### Associations of biomarkers of effect with levels of internal exposure: parameters showing an association at higher values

Some sets of effect and exposure biomarkers showed, in a bivariate regression plot, a correlation that was dependent on the range of exposure or effect and was significant after correction for confounding factors.

**Figure 2:**
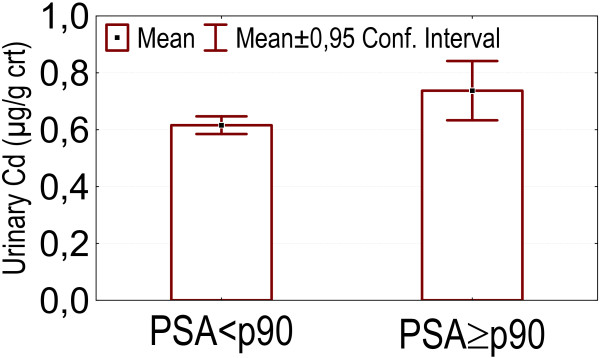
PSA levels above p90: association with higher urinary cadmium concentrations.

**Figure 3:**
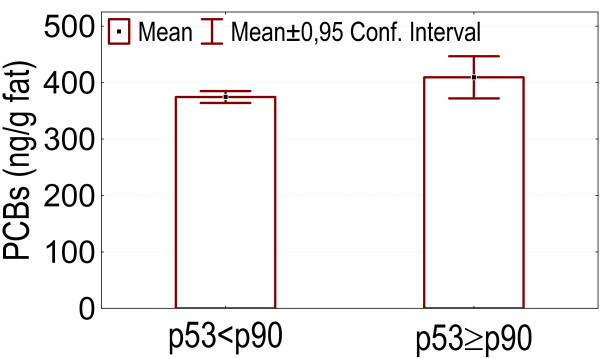
p53 levels above p90: association with higher serum marker PCB concentrations.

**Figure 4:**
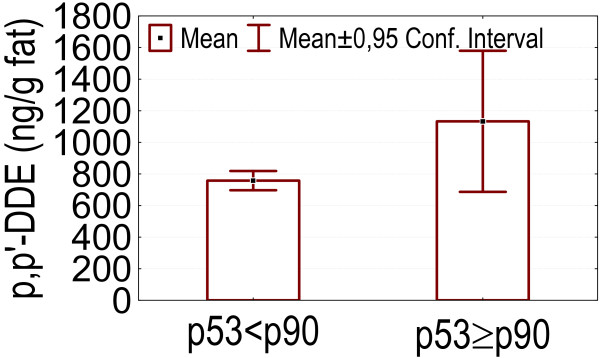
p53 levels above p90: association with higher serum p,p'-DDE concentrations.

**Figure 5:**
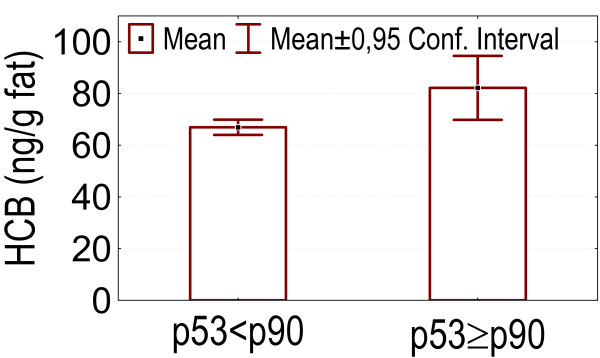
p53 levels above p90: association with higher serum HCB concentrations.

**Figure 6:**
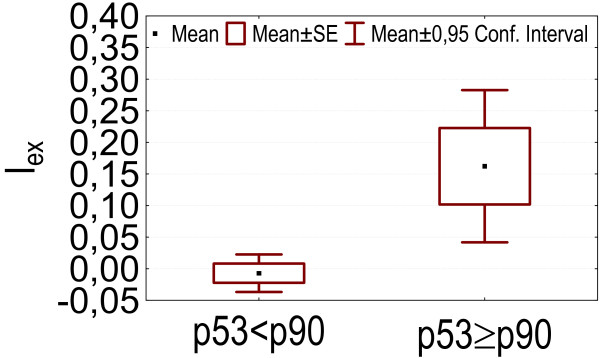
p53 levels above p90: association with higher values of the Index of Internal Exposure (I_ex_).

**Figure 7:**
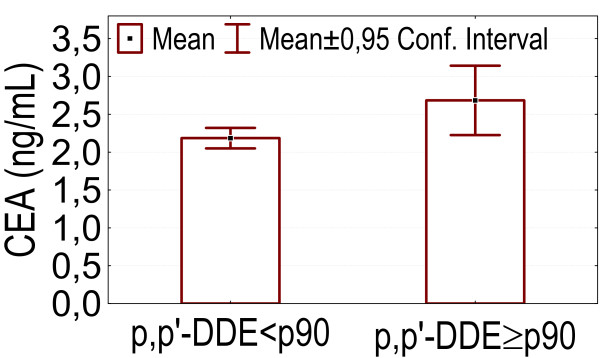
p,p'-DDE levels above p90: association with higher serum CEA levels.

**Figure 8:**
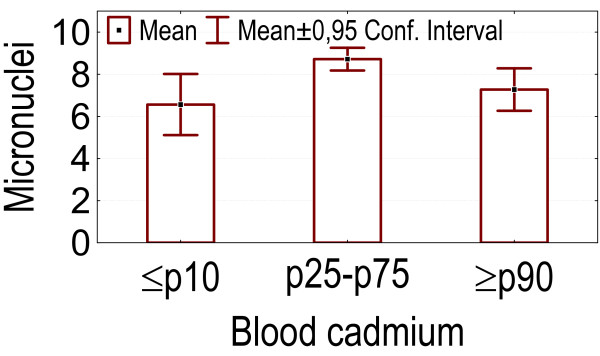
Categories of blood cadmium concentrations: association with the number of micronuclei.

**Figure 9:**
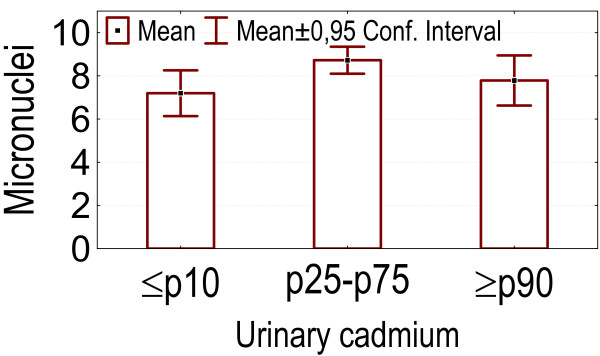
Categories of urinary cadmium concentrations: association with the number of micronuclei.

- Higher PSA levels (above p90) were associated with higher values of urinary cadmium (p = 0.029), as is shown in figure 2.

- Higher p53 levels (above p90) were associated with higher values of marker PCBs, DDE, HCB and the index of internal exposure I_ex _(p = 0.049, 0.035, 0.024, 0.00083), as is shown in figures 3, 4, 5, 6.

- Higher DDE levels (above p90) were associated with higher CEA levels (p = 0.018), as shown in figure 7.

- Higher levels of blood and urinary cadmium (above p90) were associated with lower micronuclei counts compared to the group between p25 and p75 of cadmium concentrations (p = 0.037 and 0.033 respectively). Lower levels of blood and urine cadmium (below p10) were associated with lower counts of micronuclei compared to the group with cadmium concentrations between p25 and p75, although this was not statistically significant. See figures 8, 9.

### Associations of biomarkers of effect with exposure variables derived from questionnaires

Table 6 presents relationships between exposures assessed from questionnaires and effect biomarkers. Significant positive associations were found for PSA (daily consumption of dairy fat), CEA (daily consumption of fish fat; recent alcohol consumption; average alcohol consumption, lifetime smoking, professional exposure to halogenated hydrocarbons, professional exposure to solvents), p53 level in serum (daily consumption of fruit, daily consumption of fish fat), micronuclei (daily consumption of vegetables, daily consumption of fish fat), DNA-strand breaks as measured with the comet assay (professional exposure to halogenated hydrocarbons, professional exposure to solvents, nuisance from noise during the day and from noise during the night, indoor stoking of diverse organic materials).

**Table 6:** Associations between effect biomarkers and exposure information derived from questionnaires.

**Effect biomarker**	**n**	**Parameter of exposure**	**Confounding factors**	**Regression coefficient**	**Standerdized regression coefficient (95% CI)**	**Squared semi-partial correlation**	**p-value**
PSA ng/mL (n = 770)	770	Consumption of dairy fat	A/S/LS/BMI	0.009	0.071 (0.001, 0.142)	0.005	0.047
CEA ng/mL (n = 829)	829	Lifetime smoking	A/S	0.064	0.429 (0.313, 0.546)	0.048	< 0.000001
		Consumption of Alcohol	A/S/LS	0.030	0.174 (0.110, 0.237)	0.027	< 0.000001
		Exposure to solvents	A/S/LS	0.383	0.100 (0.038, 0.161)	0.010	0.0015
		Recent alcohol consumption	A/S/LS	0.046	0.096 (0.033, 0.160)	0.008	0.0029
		Consumption of fish fat	A/S/LS	0.070	0.069 (0.008, 0.131)	0.005	0.027
		Exposure to halogenated hydrocarbons	A/S/LS	0.266	0.068 (-0.080, 0.216)	0.005	0.030
p53 pg/mL	825	Consumption of fish fat	A/S/LS	9.836	0.097 (0.028, 0.166)	0.009	0.0059
		Consumption of fruit	A/S/LS	0.115	0.098 (0.029, 0.167)	0.009	0.0056
Micronuclei (number per 1000 binucleated cells)	773	Consumption of fish fat	A/S/LS	0.312	0.108 (0.043, 0.173)	0.011	0.0011
		Consumption of vegetables	A/S/LS	0.003	0.089 (0.024, 0.153)	0.008	0.0072
DNA strand breaks (%DNA, comet assay)	593	Consumption of meat fat	A/S/RS	-0.018	-0.125 (-0.208, -0.042)	0.014	0.0033
		Indoor stoking of diverse organic materials	A/S/RS	0.228	0.111 (0.030, 0.191)	0.012	0.0072
		Exposure to solvents	A/S/RS	0.208	0.102 (0.020, 0.184)	0.010	0.015
		Consumption of freshwater fish	A/S/RS	-0.447	-0.098 (-0.178, -0.018)	0.010	0.017
		Nuisance from noise (day)	A/S/RS	0.124	0.094 (0.014, 0.175)	0.009	0.022
		Nuisance from noise (night)	A/S/RS	0.170	0.087 (0.006, 0.167)	0.007	0.036
		Exposure to halogenated hydrocarbons	A/S/RS	0.179	0.086 (0.005, 0.167)	0.007	0.039

A multiple regression was performed with each effect biomarker as dependent variable with a measured exposure variable and confounding factors as independent variables. CI: confidence interval, n: number of cases, A = age, S = sex, LS = lifetime smoking, RS = recent smoking, BMI = body mass index.

Significant negative associations were found for DNA-strand breaks as measured with the comet assay with daily consumption of meat fat and occasional consumption of self-caught freshwater fish.

**Figure 10:**
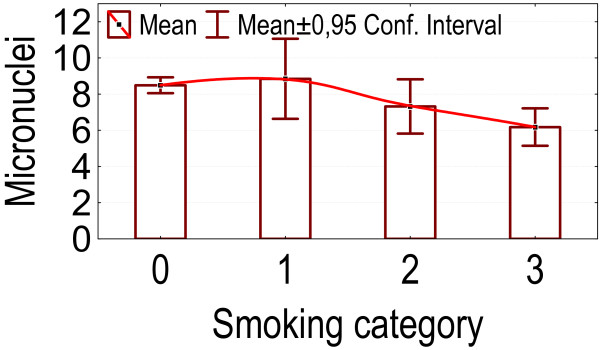
**Different smoking categories: association with the number of micronuclei**. Category 0: non-smokers; category 1: persons smoked less than 100,000 cigarettes; category 2: persons smoked at least 100,000 but less than 200,000 cigarettes; category 3: persons smoked at least 200,000 cigarettes.

Between smoking and number of micronuclei a complex relationship was observed (figure 10). Overall differences between crude values for different smoking categories were significant (p = 0.005). After correction for age and sex the overall difference is no longer significant (p = 0.09), but heavy smokers (more than 200,000 cigarettes) show significantly less micronuclei than non-smokers or light smokers (p = 0.00030 and 0.023).

Level of education was not significantly associated with effect biomarkers.

## Discussion

### Differences in association with area of residence

As in the pilot campaign of the Flemish human biomonitoring program [2,4,23] we found significant differences in effect-biomarkers in association with area of residence. Such differences were found for CEA, micronuclei, DNA-strand breaks and oxidative DNA damage.

In the 'Olen' and 'Albert Canal' industrial areas micronuclei values were significantly higher than in the area with the lowest value, suggesting that both petrochemical and non-ferro industries might contribute to the risk of cancer of people residing in their vicinity. However we found no evidence indicating that the risk of cancer of the whole population of large areas of the order of 200 km^2 ^is increased above the risk of the rest of the Flemish population by the heavy industry situated in those areas. This may in part be explained by the fact that many participants resided in zones located to the west of the industrial sites in both port areas. The dominant wind pattern in Belgium is from west to east, which means that residents living west of the point sources are less exposed. Within 'Antwerp port', the district of Burcht situated close to a large non-ferro industry showed much more DNA-strand breaks than the district of Beveren situated about 6 kilometers further to the west of the industrial sites. Our findings suggest that a detectable increase in risk might be restricted to those groups, such as the inhabitants of Burcht, residing within a few kilometers of important point sources.

Our observations indicate that residence near waste incinerators might be associated with an increase in the risk of cancer. Indeed, all three biomarkers of genotoxic effects were significantly increased, not only above the level observed in the area with the lowest value, but also above the level observed for the rest of Flanders. For each of these biomarkers the highest level was observed in residents of 'waste incinerators'. In addition, the level of CEA was above the level observed in the area with the lowest value.

Our observations also suggest that residence in cities might be associated with some increase in the risk of cancer. Both in Antwerp and in Ghent levels of CEA and of micronuclei were significantly elevated above the level observed in the area with the lowest value. In Ghent DNA strand breaks were increased compared to the level observed in the area with the lowest value, as well as above the level observed in the rest of Flanders.

Interestingly, we found no evidence that residence in an area where intensive use of pesticides occurred, increased risk of cancer. On the contrary, although the level of oxidative DNA damage observed for these residents was significantly increased compared to the level observed in the area with the lowest value, the levels of micronuclei, of DNA strand breaks, of CEA and of PSA were significantly lower than those observed for the rest of Flanders. In terms of our observations, the 'fruit area' appeared to be the area with the most favorable results.

Remarkably, results for rural Flanders were not significantly better for any of the biomarkers of effect than for the rest of Flanders. For micronuclei and DNA-strand breaks, relatively high values were observed, significantly elevated above those observed in the area with the lowest value. Correspondingly, relatively high levels of internal exposure to some pollutants were observed in residents of rural areas in Flanders in both the pilot and the subsequent biomonitoring studies in Flanders. Indeed, in the Flemish pilot biomonitoring study, women aged 50–65 residing in the rural area of Peer had higher levels of cadmium and dioxin-like activity in their blood or serum, and higher levels of cadmium and 1-hydroxypyrene in their urine than women residing in the city of Antwerp [23]. Also, adolescents residing in rural areas had blood levels of cadmium and organochlorine pollutants above Flemish reference values [5]. Concerning effect biomarkers, in the rural area of Peer men were found to have a lower sperm quality and lower testosterone levels [52], and women aged 50–65 showed higher HPRT mutant frequencies than residents of the industrial city of Antwerp. Taken together, these results indicate that, at least in some respects, internal exposure and biological effects related to environmental pollution are no less in rural areas than in other Flemish areas. We do not know how this comes about, except for the fact that certain local habits such as burning waste, inappropriate use of pesticides and consumption of self-grown food [24] could be involved. Indeed, consumption of self-grown vegetables, which is more frequent in rural areas, has been associated with a higher exposure to pesticides and cadmium and also with lower sex hormone levels and with lower sperm quality (discussed by Dhooge *et al*. [24]).

Our study was not designed to detect differences in internal exposure or in biological effects in the immediate surroundings of sources of pollution, and was only meant to observe differences between large areas with different types and levels of pollution. However, we did observe higher internal exposure (Schroijen *et al*. 2007; unpublished results) or more intense biological effects (this paper and also unpublished results on gene expression) near point sources of pollution reaching marginal or even full statistical significance, although only a low number of people were studied around these point sources. In terms of DNA strand breaks, a difference of almost a factor of three was observed. This suggests that the relatively high values of internal exposure measured in most people in Flanders, independent of their area of residence, are in part due to emissions of point sources. Although they do not lead to detectable increases in internal exposure nor to detectable biological effects in the larger area where they are located, they do lead to significantly higher internal exposure and associated biological effects in people residing at short distance. Distance from nuclear power plants showed a pronounced negative correlation with childhood leukaemia in a recent German study [53].

### Exposure effect relationships

Our results indicate that levels (see table 2) of internal exposure to some environmental pollutants as they occur in the Flemish population show indeed a positive correlation with some biomarkers of genotoxic effects and with the levels of some tumor-associated proteins. Although quite low, these levels might contribute to the relatively high risk of cancer observed in Flanders [54]. As discussed below for each of the effect biomarkers such positive correlations were observed for blood lead levels with serum levels of CEA; for urinary cadmium levels with serum levels of CEA and PSA; for urinary 1-OHP levels (a biomarker for PAH exposure) with oxidative DNA damage and serum levels of CEA; for urinary ttMA (a biomarker for benzene exposure) with oxidative DNA damage and serum levels of CEA; for serum levels of PCB 118 with the amount of DNA-strand breaks and the number of micronuclei in peripheral blood cells; for serum level of marker PCBs with p53 serum levels; for serum level of HCB with p53 serum levels; for serum level of DDE with serum levels of p53 and CEA; for an index of internal exposure with serum levels of CEA and p53.

Higher prostate specific antigen (PSA) levels (above p90) were associated with higher cadmium levels in blood. A positive association between internal exposure to cadmium and increased serum PSA values has been found previously [55-57]. Increased serum PSA levels were also found in men exposed to phenol, mixed vapours or formalin [58] and PCBs [59] and also in men with a higher intake of 2-amino-1-methyl-6-phenylimidazo [4,5-b]pyridine (PhIP), a genotoxic carcinogen formed during cooking of meat [60]. In our study, PSA-levels were also associated with dairy consumption. In the literature, positive correlations were reported between PSA and respectively dairy fat intake, total fat intake, and high calcium intake [61,62]. Higher PSA levels have been linked to an increased risk of consequently developing prostate cancer [63-65].

We found positive associations between several parameters of internal exposure and carcinoembryonic antigen (CEA) levels. This was the case for urinary cadmium, blood lead, serum DDE, urinary ttMA, urinary 1-OHP levels and for an index for internal exposure. Also smoking, alcohol consumption and consumption of fish fat, and occupational exposure to solvents or to halogenated hydrocarbons showed a positive association with serum CEA levels. In our pilot study we already found a positive association between serum CEA and an index of internal exposure based on blood levels of lead, marker PCBs and dioxin like activity, and on urinary levels of cadmium and 1-OHP [4]. Positive correlations between serum CEA levels and urinary 1-OH-P [33] and between blood levels of cadmium and CEA [66] were described previously. A positive association between CEA and smoking or alcohol consumption was reported by Verdi *et al*. [67] and Herbeth & Bagrel [68]. Also, Herbeth & Bagrel [68] reported an association between poor working conditions (noise, dust, vibrations, toxic products) and CEA levels, which is in accordance with the association we have found between CEA and occupational exposure to solvents or to halogenated hydrocarbons. CEA is a tumormarker which rises in concentration during the development of several cancers [69]. Also, elevated levels of CEA were associated with an increased risk of developing lung cancer [34] or colorectal cancer [35].

In our dataset serum levels of the tumorsuppressor protein p53 above the p90 were associated with higher levels of marker PCBs, DDE, HCB and an index of internal exposure. Howsam *et al*. [70] found a correlation between p53 gene mutations and p,p'-DDE exposure, and between PCB-exposure and colorectal cancer risk. In the pilot biomonitoring campaign in Flanders we found a positive association between blood lead and the level of anti-p53 antibodies, and also between anti-p53 antibodies and an index of internal exposure based on blood levels of lead, marker PCBs and dioxin like activity, and on urinary levels of cadmium and 1-OHP [4]. Increased p53 levels have been found in cases of exposure to other pollutants such as vinylchloride [10,14,71,72], asbestos [13] and PAHs [13,38]. Anti-p53 antibodies in sera from patients with chronic obstructive pulmonary disease can predate a diagnosis of cancer [39]. Levels of p53 can be increased in serum collected years before the clinical diagnosis in patients with asbestos or silica related occupational cancers [40,41].

Levels of micronuclei showed a positive association with higher levels of PCB 118 in serum and with a higher consumption of fish fat and vegetables. According to Park *et al*. [51] total dioxin-like PCBs are highly correlated with PCB 118 (correlation coefficient r = 0.98, p < 0.01) in human serum. The observed positive association between PCB 118 level and number of micronuclei might stem from the fact that a higher PCB 118 level reflects a higher internal exposure to dioxin-like PCBs and possibly a higher level of AHR mediated oxidative stress [73,74]. A positive correlation with the number of micronuclei in peripheral blood cells could suggest that increased PCB 118 serum levels might be associated with an increased risk of cancer, as an increased micronucleus frequency in peripheral blood lymphocytes was observed to predict the risk of cancer in humans [44]. Consistent with this, Demers *et al*. [75] found that women diagnosed with breast cancer had significantly higher serum concentrations of PCB 118 (p = 0.03) and described an association between breast cancer risk and PCB 118 serum concentration (odds ratio = 1.60, 95% confidence interval: 1.01, 2.53; fourth vs. first quartile). Nagayama *et al*. [76] found that a mixture of organochlorine compounds resembling the contamination profile present in the healthy Japanese population, efficiently induced micronuclei in human whole blood cultures. Our findings concerning PCB118 and consumption of fish fat are well compatible with the observations of Nagayama *et al*. Concerning the observed positive association between level of micronuclei and consumption of vegetables, we did not find such an association in published data. On the contrary, there are many reports on a possible protective effect of a high consumption of fruit and vegetables against DNA-damage [77]. So it is possible that our observation is a chance finding that has no implication as to the link between vegetables and health. Alternatively, it remains possible that our study population consumed vegetables contaminated by genotoxins, such as those found by Feretti *et al*. [78] in pesticide treated vegetables. Both blood and urinary cadmium levels and also smoking were associated with micronuclei in an unexpected way, with an initial (non-significant) increase in micronuclei, followed by a significant decrease at higher cadmium levels or higher levels of smoking. As to the link with smoking, Bonassi *et al*. [79] also found an unexpected association. However, contrary to our observation, they noticed an initial decrease of micronuclei formation with increased smoking frequency followed by a subsequent increase in very heavy smokers (> 30 cigarettes/day). Bonassi *et al*. [79] hypothesized that tobacco smoke may induce damage to lymphocytes, which renders them unable to survive the culture period or unable to divide. If they don't divide, they will not form binucleated cells and will not be scored for micronuclei formation. This hypothesis, applied as well to tobacco smoke as to internal exposure to cadmium, could well explain our findings.

As stated by Møller *et al*. [80] the comet assay permits detection of DNA damage in leukocytes induced by a variety of lifestyle and environmental exposures, including exercise, air pollution, sunlight, and diet. We found positive associations with PCB 118, self-reported occupational exposure to solvents or halogenated hydrocarbons, nuisance from noise during the day, nuisance from noise during the night, and indoor stoking of diverse organic materials. Negative associations were found with consumption of meat fat and self-caught freshwater fish. The positive association with serum PCB 118 level might rest, as discussed above for the induction of micronuclei, on the fact that a higher PCB 118 level reflects a higher internal exposure to dioxin-like PCBs and possibly a higher level of AHR mediated oxidative stress. The association with solvents and halogenated hydrocarbons was already observed by several authors [80,81]. Although few studies focus on the effects of noise on DNA-damage, one study reports a significant increase of DNA-damage in rat adrenal glands, which the authors hypothesise is a result of a disturbance of the redox status of the cells [82]. Burning of biomass derived fuels was found to contribute substantially to indoor air concentrations of PAH's [83]. Burning of household garbage and biomass-derived fuels were found to be important sources of PAH's and benzene [83-85]. As to the negative association of DNA strand breaks with meat fat and freshwater fish consumption, we found no similar data in the literature. On the contrary, dietary fat is considered to contribute to DNA-damage and cancer risk [77], and consumption of freshwater fish is considered to be an important source of pollutants [86,87]. So, our findings concerning meat fat and consumption of freshwater fish might be chance findings without relevance. However, consumption of freshwater sport fish might contribute to intake of protective substances such as omega-3 fatty acids and thus also confer benefits [88].

8-Hydroxy-deoxy-guanosine (HDG) results from oxidation of the guanine-residue of DNA. HDG levels were higher in subjects with a higher internal exposure to benzene and polycyclic aromatic hydrocarbons as assessed through urinary levels of ttMA and 1-OHP respectively. This was also observed by other authors [89]. Benzene and polycyclic aromatic hydrocarbons are known for their potential for inducing oxidative DNA-damage [90,91]. HDG is one of the important promutagenic lesions in relation to air pollution and lung cancer [92].

## Conclusion

Although we found the levels of genotoxic parameters and of tumor-associated proteins quite homogenous in Flanders, residence near waste incinerators, in cities, or close to important industries showed a positive correlation with biomarkers associated with carcinogenesis. Thus, residence in those areas probably contributed to the risk of cancer. Whereas for the 'fruit area' with intensive use of pesticides favourable results were obtained, in some other rural areas the levels of these biomarkers were not lower than in the rest of Flanders. In addition, we observed more intense biological effects occurring in persons residing near point sources of pollution. This suggests that the relatively high values of internal exposure measured in most people in Flanders, independent of their area of residence, are in part due to emissions of point sources, the effects of which, in terms of both internal exposure and biological effects, can only be detected in people residing at short distance. Levels of internal exposure occurring in the general population in Flanders showed positive correlations with biomarkers associated with carcinogenesis and probably contributed to the risk of cancer.

## List of abbreviations

1-OHP: 1-hydroxypyrene; ANCOVA: analysis of covariance; ANOVA: analysis of variance; BMI: body mass index; Cd: cadmium; CEA: carcinoembryonic antigen; crt: creatinin; FLEHS: Flemish Environment and Health Survey; GC-ECD: gas chromatography – electron capture detection; HCB: hexachlorobenzene; HDG: 8-hydroxy-deoxy-guanosine; HPLC: high performance liquid chromatography; ICP-MS: inductively coupled plasma – mass spectrometry; I_ex_: index of internal exposure; p53: p53 protein in serum; PAHs: polyaromatic hydrocarbons; PCB: polychlorinated biphenyls; PCDD/F: Polychlorinated Dibenzo-p-Dioxin/Polychlorinated Dibenzofuran; PLSD: Phisher protected least significant difference; PSA: prostate specific antigen; SCGE: single cell gel electrophoresis; TEQ: toxic equivalent; ttMA: t,t'-muconic acid

## Competing interests

N. van Larebeke has worked occasionally as a consultant for the World Wide Fund for Nature and presently works occasionally as a consultant for Veolia Inc, active in environmental services and waste management.

The other authors declare that they have no competing interests.

## Authors' contributions

NvL, GS, WB, VN and LB contributed to the conception and design of the study, VN, EVDM, MB, GK, MB and EDH contributed to the field work and acquisition of data, SDC and LB contributed to the analysis of the data. SDC did the literature study, SDC and NvL wrote the manuscript. All authors read and approved the final manuscript.
